# Novel perspective: exercise training stimulus triggers the expression of the oncoprotein human double minute-2 in human skeletal muscle

**DOI:** 10.1002/phy2.28

**Published:** 2013-07-15

**Authors:** Emilie Roudier, Julian Aiken, Dara Slopack, Fares Gouzi, Jacques Mercier, Tara L Haas, Thomas Gustafsson, Maurice Hayot, Olivier Birot

**Affiliations:** 1 Faculty of Health, Angiogenesis Research Group, York UniversityToronto, Canada; 2 INSERM U1046, Department of Clinical Physiology, CHRU Montpellier, University of Montpellier 1, University of Montpellier 2Montpellier, France; 3 Department of Laboratory Medicine, Section of Clinical Physiology, Karolinska Institutet, Karolinska University HospitalStockholm, Sweden

**Keywords:** FoxO1, Mdm2, PECAM-1

## Abstract

High expression levels of human double minute-2 (Hdm2) are often associated with increased risk of cancer. Hdm2 is well established as an oncoprotein exerting various tumorigenic effects. Conversely, the physiological functions of Hdm2 in nontumor cells and healthy tissues remain largely unknown. We previously demonstrated that exercise training stimulates expression of murine double minute-2 (Mdm2), the murine analog of Hdm2, in rodent skeletal muscle and Mdm2 was required for exercise-induced muscle angiogenesis. Here we showed that exercise training stimulated the expression of Hdm2 protein in human skeletal muscle from +38% to +81%. This robust physiological response was observed in 60–70% of the subjects tested, in both young and senior populations. Similarly, exercise training stimulated the expression of platelet endothelial cell adhesion molecule-1, an indicator of the level of muscle capillarization. Interestingly, a concomitant decrease in the tumor suppressor forkhead box O-1 (FoxO1) transcription factor levels did not occur with training although Mdm2/Hdm2 is known to inhibit FoxO1 expression in diseased skeletal muscle. This could suggest that Hdm2 has different targets when stimulated in a physiological context and that exercise training could be considered therapeutically in the context of cancer in combination with anti-Hdm2 drug therapies in order to preserve Hdm2 physiological functions in healthy tissues.

## Introduction

Expression or function of the tumor suppressor p53 protein is statistically altered in about 50–60% of human cancers (Soussi and Wiman 2007). The E3 ubiquitin ligase murine double minute-2 (Mdm2) is an oncoprotein mostly known for its negative regulatory role on p53 function, inhibiting p53 transcriptional activity, promoting its nuclear export, and targeting it for proteosomal degradation (Marine and Lozano 2010; Li and Lozano 2013). Interestingly, Mdm2 is often overexpressed in human cancers and can also exert some tumorigenic activity independently of p53 (Marine and Lozano 2010). For example, Mdm2 activity contributes to enhance cell proliferation and to suppress the cell cycle arrest and apoptosis by regulating various molecules such as the retinoblastoma protein, E2F transcription factors, p21, or XIAP. Also, Mdm2 involvement in promoting tumor angiogenesis and inflammation has recently emerged through its implication in vascular endothelial growth factor-A (VEGF-A) proangiogenic and NF-κB proinflammatory signaling (Nieminen et al. 2005; Carroll and Ashcroft 2008; Busuttil et al. 2010; Thomasova et al. 2012). With such broad and complex tumorigenic effects, it is not surprising that a search in the Pubmed database indicates that 90% of Mdm2-related publications refer to a tumor context.

Among current anti-cancer strategies, some aim to inhibit Mdm2 function including downregulating its expression level, inhibiting its ubiquitin ligase activity, and blocking its interaction with p53 (Vassilev 2007; Li and Lozano 2013). Clinical trials are even already in place (http://www.clinicaltrials.gov). Of importance, the administration of anti-Mdm2 drugs in animal models and even in clinical trials is usually performed with systemic delivery (Secchiero et al. 2011), raising the unanswered question of non-negligible risks of deleterious side effects for healthy tissues.

Transgenic models have clearly established that full deletion of Mdm2 is lethal (Toledo and Wahl 2006). Mendysa et al. (2006) generated Mdm2^Puro/Δ7-9^ transgenic mice harboring knockout and hypomorphic alleles for Mdm2. As a consequence these animals express only 40% of Mdm2 compared with the wild-type littermates. Mdm2^Puro/Δ7-9^ mice show no embryonic lethality and are protected against tumorigenesis.

We have recently explored further the vascular phenotype of the Mdm2^Puro/Δ7-9^ mice and demonstrated that Mdm2 plays a key physiological role in regulating rodent skeletal muscle capillarization. In sedentary Mdm2^Puro/Δ7-9^ mice, the level of muscle capillarization was decreased by 20% (Roudier et al. 2012). Interestingly, whereas prolonged endurance training stimulated Mdm2 protein expression and promoted angiogenesis in wild-type muscles, the growth of new capillaries was blunted in Mdm2^Puro/Δ7-9^ mice.

To our knowledge, this is one of very few characterizations of a physiological role for Mdm2 in an adult and healthy tissue. Skeletal muscles account for about 40% of our body weight, ensuring key functions from locomotion to metabolic regulation of glycemia. By matching the blood supply to the metabolic demand of active myofibers, the capillary network is a crucial determinant of muscle function (Egginton 2009). Thus, if inhibiting Mdm2 expression or function in tumor cells is an appealing anti-cancer strategy, we can, however, question what could be the consequence of systemic targeting for striated muscles, including respiratory muscles, diaphragm, skeletal muscles, and cardiac muscle, with an impact on vital functions such as locomotion, blood circulation, respiration, and metabolic homeostasis.

Exercise training has been established as an efficient, practical, and costless approach to minimize side effects of anti-cancer therapies in healthy tissues (Mishra et al. 2012). In the context of Mdm2, the use of rehabilitating exercise training could therefore be considered in combination with anti-Mdm2 drugs to preserve its physiological (and required) level of expression in healthy tissues.

Here, we hypothesize that the stimulatory effect of exercise training on Mdm2 muscle expression observed in rodent models will translate into human double minute-2 (Hdm2) protein, the human analog of the murine Mdm2, in human skeletal muscle.

## Methods

### Ethical approval

All research protocols conformed to the standards of the latest revision of the Declaration of Helsinki and were approved by local institutions, respectively, the ethics committee at the Karolinska Institutet (Stockholm, Sweden) for the training of young male subjects, and the ethics committee from Montpellier University Hospitals for the training of the senior population. Informed written consent was obtained from all subjects.

### Participants

Two populations of subjects were studied. Sixteen sedentary young male subjects, all healthy and without any medications, were recruited. The subjects did not undertake any regular sporting activities in the 6 months prior to the 6-week training program. These subjects were part of a larger study involving a total of 24 subjects (Keller et al. 2011). Fourteen senior subjects (seven men and seven women) aged from 50 to 75 years with no disease and less than 150 min of moderate-to-vigorous physical activity per week were recruited (Gouzi et al. 2013). The clinical characteristics of all subjects including age, body weight, height, body mass index, and peak oxygen consumption (VO_2_) are summarized in Table 1.

**Table 1:** Clinical characteristics of the young and senior populations

	Young men (*n* = 16)		Senior subjects (*n* = 14)	
Age (years)	23.4 ± 0.7		61.8 ± 1.7	
Height (cm)	179.7 ± 2.3		170.5 ± 1.9	
Body weight (kg)	73.9 ± 2.5		76.0 ± 3.0	
BMI (kg/m^2^)	22.9 ± 0.7		26.0 ± 0.7	

Peak VO_2_ (mL min^−1^ kg^−1^)	Pretraining	Posttraining*	Pretraining†	Posttraining*†
	49.7 ± 1.3	56.7 ± 1.8	25.7 ± 1.6	28.6 ± 1.6

Data are presented as means ± SEM. BMI, body mass index; Peak VO_2_, maximal oxygen consumption.

Statistical difference between pre- and posttraining conditions: *P ≤ 0.001.

Differences between young and senior subjects: †*P* ≤ 0.01.

### Exercise training protocols

Young male subjects performed an incremental cycloergometric test until exhaustion on an electrically braked cycloergometer (RE 990, Rodby innovation, Vänge, Sweden), following the individualized protocol and according to the international standards (Ross 2003). During the exercise test, heart rate, ECG, blood pressure, and transcutaneous oxygen saturation were monitored. Oxygen consumption (VO_2_) and carbon dioxide production (VCO_2_) were measured and calculated by breath-by-breath analysis (Sensormedics, Vmax 229, Autobox, Yorba Linda, CA). Maximal power output was the maximal workload sustainable, and peak oxygen consumption (peak VO_2_) was the mean value during the last 20 sec of the test. At peak VO_2_, the respiratory exchange ratio exceeded 1.10 on all occasions. The training protocol consisted of 24 sessions of 45-min cycling endurance exercise, condensed in 6 weeks, four times per week at an intensity corresponding to 70% of the pretraining peak VO_2_ (100% compliance).

Senior subjects performed an incremental cycloergometric test until exhaustion on an electrically braked cycloergometer (Ergoselect 200P, Ergolyne, Bitz, Germany), following the individualized protocol and according to the international standards (Ross 2003). During the exercise test, heart rate, ECG, blood pressure, and transcutaneous oxygen saturation were monitored. Oxygen consumption (VO_2_) and carbon dioxide production (VCO_2_) were measured and calculated by breath-by-breath analysis (Sensormedics, Vmax 229, Autobox, Yorba Linda, CA). The ventilatory threshold was blindly and independently assessed for each subject by two experienced practitioners on the basis of noninvasive methods (ventilator equivalent and V-slope methods), as recommended (Ross 2003).

The training protocol consisted of 20 sessions of 45-min cycling endurance exercise, condensed in 6 weeks, three times per week at an intensity corresponding to the subject's ventilatory threshold, and corresponding to 60 ± 5% of the pretraining peak VO_2_ (Nici et al. 2006). This intensity was continuously monitored with a cardiofrequency meter. Each session was completed by 30 min of strength building exercise (8–10 exercises, with sets of 10–15 repetitions).

All training sessions were supervised by an experienced clinician to ensure the compliance of subjects.

### Muscle biopsies

Vastus lateralis muscle biopsies were performed pretraining and 24 h after the last training session as previously described (Hayot et al. 2005; Keller et al. 2011). Muscle samples were dissected free of visible connective tissue and fat and the muscle tissue was immediately frozen in isopentane cooled to freezing point with liquid nitrogen, and stored at −80°C until analysis.

### Western blotting

Immunoblotting was carried out on protein extracts from muscle tissue as previously described (Milkiewicz et al. 2011; Roudier et al. 2012). Proteins were extracted from muscle tissue using a protein lysis buffer containing 1 mg/mL phenylmethylsulfonyl fluoride, 1 mmol/L Na_3_VO_4_, 1 mmol/L NaF (Sigma, Montreal, Canada), and 1× protease inhibitors (Complete Mini and PhosStop tablets from Roche Diagnostics, Laval, Canada). Twenty to 40 mg of frozen muscle was mixed at 4°C with lysis buffer (15 volumes of RIPA per mg of muscle). For each sample, protein extracts were prepared using two stainless carbide beads (Retsch, Fisher Scientific, Montreal, Canada) in the Retsch MM400 tissue lyser (2 × 30 pulses/sec, Retsch GmbH, Haan, Germany). Denatured samples (20–30 μg/well) were subjected to SDS-PAGE (sodium dodecyl sulfate polyacrylamide gel electrophoresis) and blotted onto nitrocellulose (Whatman, BA95, Sigma-Aldrich, Oakville, Ontario, Canada) membranes. Quality of the transfer was confirmed by Ponceau S red staining. After blocking with 5% fat-free milk at room temperature for 45 min, the blots were probed overnight at 4°C with primary antibodies against the following proteins: endothelial marker platelet endothelial cell adhesion molecule-1 (PECAM-1) (clone JC70A, cat. M0823, Dako, Burlington, Ontario, Canada), Mdm2/Hdm2 (clone 2A10, supernatant from the hybridoma previously described in Chen et al. 1993), forkhead box O-1 (FoxO1) transcription factor (clone C29H4, cat. #2880; Cell Signaling Technology, Danvers, MA), and β-actin (clone C4, cat. Sc-47778; Santa Cruz Biotechnologies, CA). β-actin was detected as a loading control. Proteins were visualized using an enhanced chemiluminescence procedure (SuperSignal West Pico, #34080; Thermo Scientific, Nepean, Ontario, Canada, or Millipore Immobilon #WBKLS0100; Thermo Scientific) and a Kodak Imaging station 4000MM Pro. Western blot images were analyzed with Carestream Molecular Imaging software. For each population of subjects, samples were randomly loaded on gels including a calibrator (i.e., loading of a protein extract sample composed of a pool of all samples) in order to conduct inter-gel comparisons.

### Statistical analysis

Analyses were performed using Prism5 software (GraphPad). Data are represented as means ± SEM. Two populations were considered: young men (*n* = 16) and senior subjects (*n* = 14). The effect of age and exercise training on the levels of expression of Hdm2, PECAM-1, and FoxO1 proteins as well as the peak VO_2_ was analyzed using a two-way analysis of variance (ANOVA) followed by Bonferroni posttests. Results were considered statistically significant when *P* ≤ 0.05.

## Results

### Exercise training improves fitness level and increases muscle endothelial content

The maximal oxygen consumption (peak VO_2_) is a good indicator of the cardiovascular fitness level. Young subjects presented higher pretraining and posttraining levels of peak VO_2_ than the seniors (Table 1, pretraining: 49.7 ± 1.3 vs. 25.7 ± 1.6 mL kg^−1^ min^−1^ [+93%], posttraining: 56.7 ± 1.8 vs. 28.6 ± 1.6 mL kg^−1^ min^−1^ [+98%], *P* < 0.001). Exercise training increased the peak VO_2_ similarly in young and senior subjects (respectively, +14% and +11%, *P* < 0.01).

The level of muscle vascularization is considered to be an important determinant of exercise capacity (Wagner 2010) and we have previously shown that the level of expression of the PECAM-1 protein reflects the level of rodent skeletal muscle vascularization (Roudier et al. 2009, 2012).

**Figure 1:**
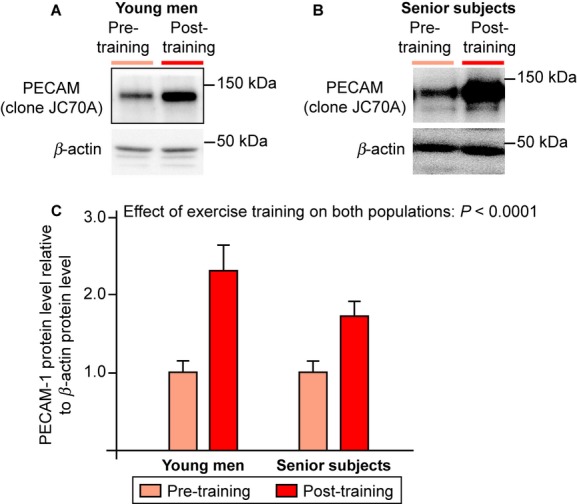
Exercise training increases expression of the endothelial marker platelet endothelial cell adhesion molecule-1 (PECAM-1) in human skeletal muscle. (A and B) Representative immunoblots of PECAM-1 protein expression in the vastus lateralis muscle from young men (A, n = 16) or senior subjects (B, n = 14) before and after endurance training. C, Densitometric analysis of PECAM-1 protein expression is represented and β-actin was used as a loading control. Data are presented as means ± SEM. The effect of exercise training or age was considered statistically significant when P ≤ 0.05 after two-way ANOVA analysis and Bonferroni posttest.

Representative immunoblots for PECAM-1 pre- and posttraining expression levels in skeletal muscles from young and senior subjects are shown on Figure 1A and B. Following exercise training, PECAM-1 protein expression in skeletal muscle was increased in both populations, respectively, by 129% in young men and 72% in the senior subjects (Fig. 1C, 1.00 ± 0.15 vs. 2.30 ± 0.34 arbitrary units in young men [*n* = 16]; 1.00 ± 0.14 vs. 1.72 ± 0.20 arbitrary units in senior subjects [*n* = 14], *P* < 0.0001).

Although the group of young subjects was only comprised of men, stratified analyses did not show any evidence of differences between senior men and women so results are pooled across gender for the senior group. No effect of age was observed between young and senior populations.

### Hdm2 protein expression in skeletal muscle increases with exercise training independently of age

**Figure 2:**
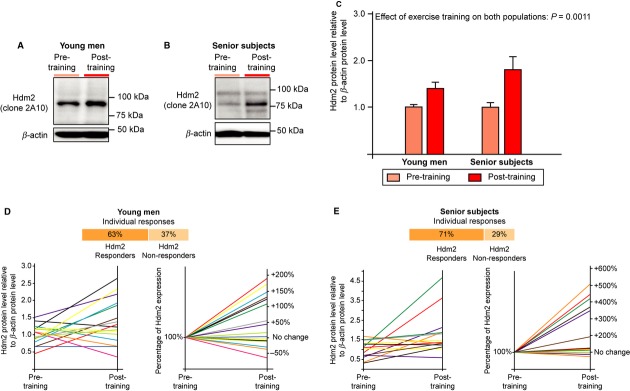
Exercise training increases expression of human double minute-2 (Hdm2) protein in human skeletal muscle. (A and B) Representative immunoblots of Hdm2 protein expression in the vastus lateralis muscle from young men (A, n = 16) or senior subjects (B, n = 14) before and after endurance training. C, Densitometric analysis of Hdm2 protein expression is represented and β-actin was used as a loading control. Data are presented as means ± SEM. The effect of exercise training or age was considered statistically significant when P ≤ 0.05 after two-way ANOVA analysis and Bonferroni posttest. (D and E) Representation of individual responses to training for Hdm2 protein expression in muscles from young (D, n = 16) and senior subjects (E, n = 14). Individual responses are expressed as raw values (Hdm2 normalized to β-actin, top graph) and in percentage of change from pretraining (bottom graph). For each population, the percentages of Hdm2 responders (i.e., subjects having an increased expression of Hdm2 in response to training) and nonresponders are indicated.

Representative immunoblots for Hdm2 pre- and posttraining expression levels in skeletal muscles from young and senior subjects are shown on Figure 2A and B. Hdm2 protein expression was significantly increased by 38% in response to exercise training in young male subjects and by 81% in the senior population (Fig. 2C, 1.00 ± 0.07 vs. 1.38 ± 0.16 arbitrary units in young men [*n* = 16]; 1.00 ± 0.11 vs. 1.81 ± 0.29 in senior subjects [n = 14], *P* = 0.0011). No effect of age or gender was detected, respectively, between young and senior populations or between senior men and senior women.

Analysis of individual Hdm2 variations in response to exercise training revealed similar proportions of Hdm2 responders (i.e., Hdm2 increase posttraining) between young and senior subjects (respectively, 63% and 71%, Fig. 2D and E).

### Exercise training had no effect on protein expression level of Hdm2 target FoxO1

**Figure 3:**
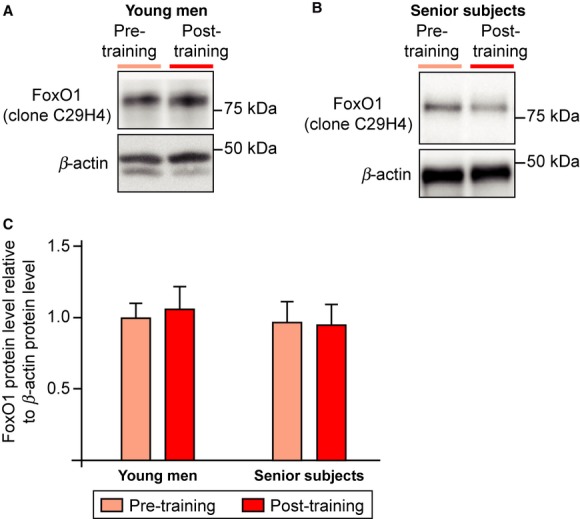
Forkhead box O-1 (FoxO1) transcription factor expression is not affected by exercise training in human skeletal muscle. (A and B) Representative immunoblots of FoxO1 protein expression in the vastus lateralis muscle of young men (A, n = 16) or senior subjects (B, n = 7 men and n = 7 women) before and after endurance training. Densitometric analysis of FoxO1 protein expression is represented and β-actin was used as a loading control. No significant difference was observed in response to exercise training (two-way ANOVA and Bonferroni posttests).

In either young or senior subjects, exercise training had no significant effect on FoxO1 protein expression (Fig. 3, young men: 1.00 ± 0.10 pretraining vs. 1.06 ± 0.16 posttraining; senior subjects: 1.00 ± 0.16 pretraining vs. 0.98 ± 0.16 posttraining).

## Discussion

Our study identified exercise training as a stimulator of Hdm2 expression in human skeletal muscle. This is a robust physiological response that occurs in 60–70% of the population independently of age and gender. It also illustrates that our previous finding of a stimulatory effect of exercise training on Mdm2 levels in rodent muscle translate to humans.

Concomitantly with the increase in Hdm2, exercise training also stimulated the expression of PECAM-1, a good indicator of the level of endothelial material (Roudier et al. 2009, 2012). This finding is in line with our previous study identifying Mdm2 as an important regulator of skeletal muscle capillarization in Mdm2^Puro/Δ7-9^ mice and showing that the reduction in Mdm2 expression restrained the proliferative and migratory response of skeletal muscle endothelial cells to the exercise stimulus (Roudier et al. 2012).

Demonstrating that exercise stimulus increases Hdm2 protein levels in human skeletal muscle might have important clinical consequences. Hdm2 is considered as an oncoprotein based on its various tumorigenic functions, and particularly its role as the main negative regulator of the tumor suppressor p53 (Li and Lozano 2013). The development of Mdm2/Hdm2 inhibitors is a very attractive approach to restore p53 function in cancer cells and to inhibit Mdm2-mediated tumor angiogenesis (Vassilev 2007; Millard et al. 2011 Li and Lozano 2013). In fact, clinical trials are already under development (Vassilev 2007; Millard et al. 2011).

The ability to stimulate Hdm2 muscle expression using rehabilitating exercise training might thus represent an easy and practical approach to preserve Hdm2 function in skeletal muscles from cancer patients undergoing anti-Hdm2 therapy. In particular, Hdm2 response to exercise training was as strong in the senior population, equally composed of men and women and trained at a very moderate intensity, as in young male subjects. The ability of the exercise training stimulus to increase Hdm2 muscle expression is therefore independent of the age, the gender, and the level of activity of the subjects, which makes the concept of combining anti-Hdm2 cancer therapies with rehabilitating exercise training very realistic. In line with this idea, physical activity is known to improve the overall quality of life and to decrease fatigue in cancer patients (Mishra et al. 2012), whereas most current anti-cancer therapies can unfortunately induced damages in non-cancer cells (Ballard-Barbash et al. 2012). Therefore, triggering Hdm2 expression by exercise training in healthy tissues might have strong antiapoptotic and survival effects in non-cancer cells, protecting them from anti-cancer therapy side effects.

Aside from the context of cancer, Hdm2 could also represent a new therapeutic target to stimulate skeletal muscle angiogenesis in chronic metabolic diseases associated with capillary regression such as obesity, diabetes, and limb ischemia. This is consistent with our previous works showing some alterations of Mdm2 expression or activation in rodent models of type-2 diabetes and limb ischemia (Milkiewicz et al. 2011; Roudier et al. 2012).

A balance between pro- and antiangiogenic factors tightly controls muscle angioadaptation, that is, the process regulating capillary maintenance, regression, or growth (Olfert and Birot 2011). Interestingly, Hdm2/Mdm2 could potentially affect both sides of the angioadaptive balance. Mdm2 was suggested to enhance the expression of the proangiogenic VEGF-A (Nieminen et al. 2005; Carroll and Ashcroft 2008). We recently showed in skeletal muscles from Mdm2^Puro/Δ7-9^ mice that Mdm2 was indispensable for the increase expression of VEGF-A in response to acute exercise (Roudier et al. 2012). Mdm2 could also contribute to restrain p53-mediated antiangiogenic effects. For example, p53 stimulates the expression of thrombospondin-1 (TSP-1) (Dameron et al. 1994), a key antiangiogenic regulator of muscle angioadaptation (Olfert and Birot 2011). Interestingly, Mdm2 could also regulate TSP-1 independently of p53. We have shown that Mdm2^Puro/Δ7-9^ mice express higher muscle levels of a disintegrin and metalloproteinase with thrombospondin motifs 1 (ADAMTS-1) expression (Roudier et al. 2012), a protein enhancing TSP-1 cleavage and promoting its antiangiogenic activity (Lee et al. 2006).

Several other angioadaptive molecules might be under the control of Mdm2 such as the FoxO1 transcription factor that is known to exert antiangiogenic effects (Milkiewicz et al. 2011; Roudier et al. in press) and to be negatively regulated by Mdm2 (Fu et al. 2009). FoxO1 is also considered a tumor suppressor (Arden 2007). Interestingly, in this study, the increase in Hdm2 expression in trained human skeletal muscle was not accompanied by a decrease in FoxO1. This suggests that an increase in Hdm2 in a healthy tissue in response to a physiological stress might not be associated with an increase in its oncogenic function.

In summary, our results showed that Hdm2 expression in human skeletal muscle was increased in response to exercise training concomitantly with higher levels of capillarization. This observation provides new insight into the mechanisms by which physical activity might improve muscle function. Further studies are required to investigate the underlying mechanisms by which exercise training modulates Hdm2 and its targets. A better understanding of the molecular events that regulate Hdm2 in nonpathological versus oncogenic contexts might contribute to the development of new anti-Hdm2 drugs that are more efficient and less deleterious to healthy tissues, and thus limiting potential side effects of these promising anti-cancer therapies.
